# Some of "Ierne's" Questions Answered?

**Published:** 1918-11-02

**Authors:** 


### THE EDITOR'S LETTER-BOX.

**[Correspondence on all subjects is invited, but we cannot in any way be responsible for the opinions expressed by our correspondents, who must give their name and address as a guarantee of good faith, but not necessarily for publication. Correspondents are reminded that brevity of style and conciseness of statement greatly facilitate early insertion.]**

### Some of "Ierne's" Questions Answered?

*To the Editor of* The Hospital.

Sir,—I regret that I have been too busy to reply to "Ierne's" questions in time for publication last week. (1) The committee deprives the matron of a "free hand." When I say the committee I mean to include its sub-divisions—*e.g.*, house committee, finance committee, medical men's committee, etc. The members of all but the latter are laymen. The matron is *employed by them*, and therefore is not in an independent position. The matron who did not consider the wishes of her committee, or who tried to work against them, would be "requested to resign." Resignation is a serious thing for a matron, and she would find it a very much more difficult matter to get another matronship than a sister or nurse has to get another post. Thus, however much a matron may wish for reforms, if her committee is obstinate she can do nothing.

(2) I confess to not understanding the purport of this question.

(3) The British public do not appear to have ever given much thought to the matter. This is a sphere in which "Ierne" might do good work. May I suggest that he (?) should devote his energies to impressing upon the public the high value of the nurse instead of using them to spread unrest and discontent among the members of the nursing profession? I verily believe some persons think a nurse's knowledge is limited to bedmaking! Although "Ierne" is probably not qualified to inform the public of all the theoretical and practical studies demanded of a nurse, he (?) can perhaps help to raise the public's opinion of her status.

(4) The chief "tenet" of our profession is *loyalty*, which precludes disapproval or criticism of those in authority. Discipline is necessary to the working of a hospital, and it is certainly against our "tenets" to undermine that discipline by disloyal grumbling.

Any complaint may be made by any member of the staff to the matron, and will, in the majority of cases, be investigated without prejudice, and justice meted out to whatever persons concerned.

The complaint I have to make against some of "Ierne's" words is that they are *inciting to disloyalty*.

(5) It is *not* "disloyalty to high ideals which seeks to raise the status of the profession so that the best, rather than the second best, class of recruits shall seek entrance," but, as presumably the incentive for these desirable candidates to enter the profession is to be higher pay, shorter hours, etc., why not tackle the laymen's committees, instead of trying to sow discontent among the present-day nurses?

By all means let us have well-educated, high-principled recruits for the future. It rests with hospital committees, backed by public support, to find the funds for larger nurses' homes, increase of staff, higher salaries. Let the reform be carried out by a public appreciative of the nurses' worth, but do not suggest to nurses that they should force the public's hand by a series of undignified agitations.

In conclusion, I refuse to "hang up the halo." The "halo," *i.e.* love of nursing for its own sake, the ideal of helping suffering humanity, is responsible for the conversion of the nurse from the "Sairey Gamp" type to the "Florence Nightingale" one.—Yours faithfully,

